# The interplay between teamwork, clinicians’ emotional exhaustion, and clinician-rated patient safety: a longitudinal study

**DOI:** 10.1186/s13054-016-1282-9

**Published:** 2016-04-19

**Authors:** Annalena Welp, Laurenz L. Meier, Tanja Manser

**Affiliations:** Department of Psychology, University of Fribourg, Rue Faucigny 2, 1700 Fribourg, Switzerland; Institute of Work and Organizational Psychology, University of Neuchâtel, Neuchâtel, Switzerland; Institute for Patient Safety, University Hospital Bonn, Stiftsplatz 12, 53111 Bonn, Germany; ETH Zurich, Department of Management, Technology and Economics, Weinbergstrasse 56/58, 8092 Zurich, Switzerland

**Keywords:** Healthcare team, Interprofessional teams, Teamwork, Emotional exhaustion, Patient safety, Intensive care

## Abstract

**Background:**

Effectively managing patient safety and clinicians’ emotional exhaustion are important goals of healthcare organizations. Previous cross-sectional studies showed that teamwork is associated with both. However, causal relationships between all three constructs have not yet been investigated. Moreover, the role of different dimensions of teamwork in relation to emotional exhaustion and patient safety is unclear. The current study focused on the long-term development of teamwork, emotional exhaustion, and patient safety in interprofessional intensive care teams by exploring causal relationships between these constructs. A secondary objective was to disentangle the effects of interpersonal and cognitive-behavioral teamwork.

**Methods:**

We employed a longitudinal study design. Participants were 2100 nurses and physicians working in 55 intensive care units. They answered an online questionnaire on interpersonal and cognitive-behavioral aspects of teamwork, emotional exhaustion, and patient safety at three time points with a 3-month lag. Data were analyzed with cross-lagged structural equation modeling. We controlled for professional role.

**Results:**

Analyses showed that emotional exhaustion had a lagged effect on interpersonal teamwork. Furthermore, interpersonal and cognitive-behavioral teamwork mutually influenced each other. Finally, cognitive-behavioral teamwork predicted clinician-rated patient safety.

**Conclusions:**

The current study shows that the interrelations between teamwork, clinician burnout, and clinician-rated patient safety unfold over time. Interpersonal and cognitive-behavioral teamwork play specific roles in a process leading from clinician emotional exhaustion to decreased clinician-rated patient safety. Emotionally exhausted clinicians are less able to engage in positive interpersonal teamwork, which might set in motion a vicious cycle: negative interpersonal team interactions negatively affect cognitive-behavioral teamwork and vice versa. Ultimately, ineffective cognitive-behavioral teamwork negatively impacts clinician-rated patient safety. Thus, reducing clinician emotional exhaustion is an important prerequisite of managing teamwork and patient safety. From a practical point of view, team-based interventions targeting patient safety are less likely to be effective when clinicians are emotionally exhausted.

## Background

In recent years, the significance of effective teamwork for the provision of safe, high-quality care in fast-paced, unpredictable environments such as intensive care has been increasingly recognized [1]. Teamwork generally comprises cognitive, behavioral, and interpersonal processes [2–4]. Interpersonal teamwork such as the quality of collaboration between nurses and physicians is considered the foundation upon which team cognitions and behaviors unfold [2]. While interprofessional teamwork has been shown to contribute to improved patient outcomes in low-acuity care settings, evidence on the role of interprofessional teamwork in intensive care is only beginning to emerge [5, 6]. Further, cognitive-behavioral teamwork such as the extent to which team members share a representation of care tasks or the ability to communicate about and jointly execute this task have also been shown to be associated with patient safety [7, 8].

Nurses and physicians who are dissatisfied with the quality of teamwork in their unit experience more emotional exhaustion [9, 10]. In acute care, about one third of clinicians are affected by it [11]. Moreover, emotional exhaustion is associated with patient safety indicators such as errors and adverse events, and should therefore be a particular concern in settings such as intensive care where consequences of errors are more severe [12].

Previous research has investigated cross-sectional relationships between either two of the three concepts. Thus, it is not known how teamwork, clinician emotional exhaustion, *and* patient safety are causally related [7, 10, 13]. For instance, it is not clear whether effective teamwork prevents clinician emotional exhaustion, or whether emotionally exhausted clinicians possess fewer resources to contribute to effective teamwork. Further, the differential role of interpersonal and cognitive-behavioral teamwork in relation to clinician emotional exhaustion and patient safety is unclear [14, 15].

This longitudinal study examines causal effects between interpersonal and cognitive-behavioral teamwork, emotional exhaustion, and patient safety in interprofessional intensive care teams. We propose that interpersonal and cognitive-behavioral teamwork have a positive effect on clinician-rated patient safety, and that they reduce clinician emotional exhaustion. In addition, we assume that clinician emotional exhaustion decreases clinician-rated patient safety. Knowledge of causal relationships will generate valuable knowledge on the management of intensive care teams and for improving clinician and patient outcomes.

## Methods

### Participants and procedures

The study was conducted in intensive care units (ICUs) across all language regions in Switzerland. To investigate causal effects, longitudinal data were collected from medical and nursing staff using an online survey that included three assessments at 3-month intervals. We contacted nursing and medical leaders of each unit, informing them about the purpose of the study and asking them to discuss participation within their teams. Unit leaders of participating units were sent a link to the survey and forwarded it to their team.

Out of the 81 ICUs in Switzerland, 55 chose to participate (67 %). The ICUs comprised all specialties (medical, surgical, pediatric, and mixed) and were distributed across 48 hospitals. Participants were 2100 nurses and physicians (month 1: *n* = 1460; month 4: *n* = 1007; month 7: *n* = 807; *n* = 493 clinicians participated at all three measurement occasions). Nurses comprised registered nurses (RNs), registered nurses with specialization in intensive care, registered nurses in training for intensive care specialization, and nurse unit managers. Physicians specialized in intensive care, anesthesia, surgery, pediatrics, internal medicine, or trauma medicine and included resident physicians, senior physicians, and head physicians (see Table 1 for descriptive statistics).

**Table 1:** Descriptive statistics

	Month 1	Month 4	Month 7
Unit level			
	*Frequency (percentage)*		
Type of hospital (university/cantonal/regional)	4 (8)/11 (23)/32 (67)		
Type of ICU (medical/surgical/pediatric/mixed)	5 (10)/7 (13)/2 (4)/41 (75)		
	*Mean (range)*		
Beds	12 (6–40)		
Patients treated per month	90 (10–405)	80 (18–314)	87 (24–447)
Individual level			
	*Frequency (percentage)*		
N	1460	1007	807
Gender (male/female)	1028 (70.4)/365 (25.0)	755 (75.0)/250 (24.8)	583 (72.2)/218 (27.0)
Professional role (nurse/physician)	1131 (77.5)/243 (16.6)	506 (50.2)/90 (8.9)	357 (44.2)/72 (8.9)
Head RNs	75 (5.1)	7 (0.7)	4 (0.5)
Intensive care RNs	779 (53.4)	114 (11.3)	82 (10.2)
RNs in intensive care training	116 (7.9)	16 (1.6)	20 (2.5)
Head physicians	70 (4.8)	7 (0.7)	7 (0.9)
Senior physicians	85 (5.8)	25 (2.5)	25 (3.1)
Resident physicians	77 (5.3)	20 (2.0)	33 (4.1)
Workload in hospital (full time/part time)	684 (46.8)/703 (48.2)	443 (44.0)/527 (52.3)	334 (41.4)/446 (55.3)
Workload in ICU (full time/part time)	683 (46.8)/700 (47.9)	443 (44.0)/527 (52.3)	201 (24.9)/379 (47.0)
	*Mean (standard deviation)*		
Age	39.56 (9.33)	40.44 (9.33)	40.64 (9.07)
Tenure (years in organization)	9.80 (8.35)	8.29 (7.59)	7.22 (6.71)
Tenure (years in ICU)	8.21 (7.69)	6.53 (7.01)	5.35 (6.23)
Professional experience	12.57 (8.94)	11.57 (8.71)	10.30 (8.50)
Cognitive-behavioral teamwork	5.24 (0.81)	5.25 (0.76)	5.21 (0.78)
Interpersonal teamwork	3.13 (0.61)	3.14 (0.63)	3.11 (0.62)
Emotional exhaustion	2.73 (0.84)	2.67 (0.83)	2.65 (0.85)
Clinician-rated patient safety	3.71 (0.62)	3.71 (0.59)	3.70 (0.59)

Not all units and participants provided all demographic information at all measurement occasions. N = 493 clinicians participated across all three measurement occasions

*ICU* intensive care unit, *RN* registered nurse

To investigate the potential impact of attrition, differences on study variables were tested between participants who completed the month 7 assessment and participants who dropped out of the study before month 7 (see Table 2). Participants who completed the month 7 assessment were slightly older, had more professional experience and longer tenure. They also reported lower emotional exhaustion. This phenomenon is commonly found in longitudinal studies assessing aspects of work stress and interpreted as dropout of highly stressed individuals. We found no significant group differences for the other variables included in the statistical model. Thus, it is unlikely that changes in the sample structure influenced study results – if anything, the effects of emotional exhaustion might have been more pronounced.

**Table 2:** Comparison of participants of month 1 and/or month 4 to participants of all three measurement occasions

	Month 1 and/or 4		Month 1, 4, 7			
	*Frequency*				*df*	*Chi square*
Gender (male/female)	1280/278		287/102		1	0.43
Professional role	1271/441		159/18		1	0.71^**^
	*M*	*SD*	*M*	*SD*	*df*	*t value*
Age	38.80	9.44	41.03	9.02	1408	-4.12^***^
Tenure (years in organization)	9.00	8.05	11.04	8.76	1404	-4.16^***^
Tenure (years in ICU)	7.30	7.44	9.69	7.87	1408	-5.29^***^
Professional experience	11.62	8.99	14.16	8.51	1418	-4.84^***^
Cognitive-behavioral teamwork month 1	5.23	0.83	5.27	0.77	1347	-0.87
Interpersonal teamwork month 1	3.11	0.63	3.14	0.59	1332	-0.81
Emotional exhaustion month 1	2.79	0.86	2.60	0.79	1310	3.88^***^
Clinician-rated patient safety month 1	3.71	0.65	3.70	0.58	1307	0.42
Cognitive-behavioral teamwork month 4	5.22	0.80	5.28	0.74	935	-1.27
Interpersonal teamwork month 4	3.14	0.63	3.13	0.63	927	0.002
Emotional exhaustion month 4	2.75	0.85	2.56	0.79	908	3.33^***^
Clinician-rated patient safety month 4	3.72	0.61	3.70	0.56	908	0.60

Chi square: dichotomous variables; *t* tests: continuous variables. Workload in hospital/ICU were not included in analyses because the number of clinicians who indicated their workload across all measurement occasions was insufficient

*M* mean, *SD* standard deviation, *ICU* intensive care unit

^**^ *p* < .01 (two-tailed test); ^***^ *p* < .001 (two-tailed test)

### Measures

Reliability statistics for all measures at all measurement occasions are reported in Table 3.

**Table 3:** Correlations between study variables

			1	2	3	4	5	6	7	8	9	10	11	12
Month 1	1	Professional role												
	2	Cognitive-behavioral teamwork	.18^***^	(.89)										
	3	Interpersonal teamwork	.24^***^	.49^***^	(.86)									
	4	Emotional exhaustion	.01	-.26^***^	-.23^***^	(.87)								
	5	Clinician-rated patient safety	.14^***^	.49^***^	.38^***^	-.25^***^								
Month 4	6	Cognitive-behavioral teamwork	.14^***^	.69^***^	.45^***^	-.19^***^	.40^***^	(.89)						
	7	Interpersonal teamwork	.21^***^	.48^***^	.66^***^	-.23^***^	.31^***^	.51^***^	(.87)					
	8	Emotional exhaustion	.05	-.19^***^	-.20^***^	.81^***^	-.16^***^	-.22^***^	-.23^***^	(.88)				
	9	Clinician-rated patient safety	.12^***^	.44^***^	.33^***^	-.18^***^	.56^***^	.46^***^	.35^***^	-.25^***^				
Month 7	10	Cognitive-behavioral teamwork	.17^***^	.71^***^	.43^***^	-.16^***^	.37^***^	.74^***^	.48^***^	-.13^***^	.41^***^	(.90)		
	11	Interpersonal teamwork	.26^***^	.42^***^	.63^***^	-.16^***^	.21^***^	.45^***^	.68^***^	-.15^***^	.31^***^	.48^***^	(.89)	
	12	Emotional exhaustion	.05	-.16^***^	-.23^***^	.75^***^	-.11^*^	-.13^**^	-.13^***^	.83^***^	-.19^***^	-.22^***^	-.24^***^	(.88)
	13	Clinician-rated patient safety	.12^*^	.35^***^	.23^***^	-.18^***^	.48^***^	.42^***^	.33^***^	-.17^***^	.57^***^	.48^***^	.32^***^	-.18^***^

Cronbach’s alphas for each scale in brackets

^*^ *p* < .05 (two-tailed test); ^**^ *p* < .01 (two-tailed test); ^***^ *p* < .001 (two-tailed test)

#### Teamwork

Teamwork was assessed with two scales covering cognitive-behavioral and interpersonal aspects of teamwork.

#### Cognitive-behavioral teamwork

We measured the cognitive-behavioral aspect of teamwork with the validated German, French, and Italian translations of the nine-item safety organizing scale, which was originally developed as a safety culture scale [16, 17]. However, it essentially measures aspects of a team’s organizing and coordination behaviors and underlying cognitions on the *team* level. For instance, it covers team cognitions and behaviors such as knowledge about and utilization of collective expertise (sample item: “We have a good map of each other’s talents and skills”). Responses are given on a 7-point Likert scale (1 = *not at all*, 7 = *to a very great extent*).

#### Interpersonal teamwork

We measured the interpersonal aspect of teamwork with the validated German, French, and Italian translations of the three-item nurse-physician relationship scale from the nursing work index revised (NWI-R) [18–20]. The scale assesses clinicians’ perception of teamwork quality between nurses and physicians (sample item: “Physicians and nurses have good working relationships”). Responses are given on a 4-point Likert scale (1 = *disagree*, 4 = *agree*).

#### Emotional exhaustion

We measured clinician emotional exhaustion with the validated German, French, and Italian translations [21–23] of the emotional exhaustion subscale of the Maslach Burnout Inventory-Human Services Survey (MBI-HSS) [24]. Emotional exhaustion is the core dimension of burnout, and it is characterized by feeling fatigued, emotionally drained, and lacking the energy to face work-related tasks (sample item: “I feel mentally exhausted because of my work”) [25]. Responses are given on a 6-point Likert scale (1 = *never*, 6 = *very often*).^1^

#### Clinician-rated patient safety

We measured clinician-rated patient safety with a one-item scale from the validated German, French, and Italian translations of the Hospital Survey of Patient Safety Culture (HSOPSC) [26–29]. Clinicians were asked to rate patient safety on their unit (“Please give your unit in this hospital an overall grade on patient safety”). Responses are given on a 5-point Likert Scale (1 *= unsatisfactory,* 5 *= excellent*).

To examine how representative individual safety ratings are of general perception of safety in each unit, we measured the agreement on patient safety per unit by calculating within-group interrater reliability (r_WG_) values [30]. The r_WG_ index compares the standard deviation (SD) of raters on each unit to the standard deviation that was to be expected if ratings were completely at random. The values ranged from .50 to .94, with a mean of .81 (*SD* = .17), indicating that there was a high level of agreement regarding overall safety between clinicians in each unit.

#### Covariates

Potential differences between professions in perceptions of teamwork, emotional exhaustion, and clinician-rated patient safety were taken into account by controlling for professional role (nurse/physician).

### Analyses

#### Hypothesis testing

**Fig. 1:**
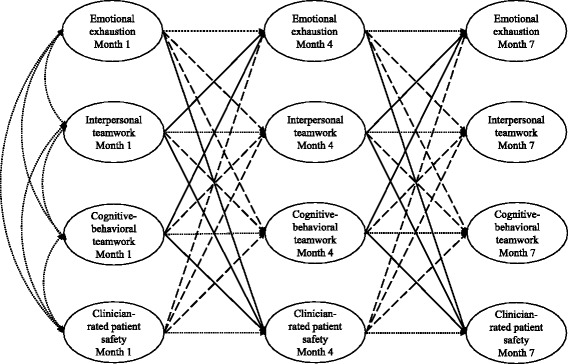
Cross-lagged structural equation model testing longitudinal relationships between teamwork, clinician emotional exhaustion, and patient safety. *Oblique solid arrows*: hypothesized cross-lagged effects between measurement occasions. *Oblique dashed arrows*: reverse cross-lagged effects between measurement occasions. *Horizontal arrows*: auto-regressive paths of the same variable between different measurement occasions. *Curved arrows*: shared variance among the predictors

We tested our hypotheses by conducting structural equation modeling (SEM) analyses using Mplus version 7 [31]. This statistical method analyzes patterns of covariance (i.e., how much two or more variables change together) within a network of variables. To test causal relationships between variables, a cross-lagged design was used (see Fig. 1). Compared to cross-sectional data, which can only yield information on *associations* between variables at the *same time* and hence is mute about the causal direction, cross-lagged models take advantage of longitudinal data by estimating the effect of a predictor at an earlier time point on an outcome at a later time point.

Of particular importance, by simultaneously estimating the hypothesized causal effect (e.g., interpersonal teamwork at month 1 predicts emotional exhaustion at month 4; *cross-lagged effect;* oblique solid arrows in Fig. 1) as well as the alternative causal effect (e.g., emotional exhaustion at month 1 predicts interpersonal teamwork at month 4; *reverse cross-lagged effect*; oblique dashed arrows in Fig. 1), cross-lagged models inform us about the causal direction of the studied effects.

In addition to estimating the hypothesized cross-lagged and reverse cross-lagged effects, the potentially confounding effect of each variable’s temporal stability was taken into account by controlling for the baseline level of the variables across time (e.g., the effect of emotional exhaustion at month 1 on emotional exhaustion at month 4; *autoregressive effect*, horizontal arrows in Fig. 1).

Furthermore, the predictors at month 1 were correlated to account for shared variance among them (i.e., the extent to which variations in the predictors overlap; curved arrows in Fig. 1). To account for variance due to measurement occasion (i.e., variance that is not explained by the cross-lagged effects), we cross-sectionally correlated the residual variances at month 4 and month 7 (not depicted in Fig. 1 to increase readability).

Finally, we calculated the average of parallel effects between month 1 and month 4, and month 4 and month 7 (e.g., interpersonal teamwork at month 1 predicting emotional exhaustion at month 4, and interpersonal teamwork at month 4 predicting emotional exhaustion at month 7). This approach identifies only stable effects as significant, and adjusts large effect sizes, thus reducing the complexity of the model and increasing precision and generalizability of results.

#### Model estimation and fit

Full information maximum likelihood (FIML) estimation for complex survey data was applied. This approach incorporates all available data into the statistical analyses, thus including individuals who did not respond at all measurement occasions, or did not complete the survey at one measurement occasion. It yields more reliable results compared to traditional methods of handling missing data, such as pairwise or listwise deletion, which excludes individuals from analyses if their survey responses are incomplete and thus reduces statistical power.

We furthermore accounted for the clustered data structure: in this sample, clinicians are clustered within their teams. Data obtained from members of the same team are not independent [32]. Ignoring clustered data structures can lead to underestimation of standard errors of effects and thus an overestimation of statistical significance. Data clustering was taken into account by adjusting the standard error [33].

## Results

Based on an outlier analysis following best-practice recommendations we deleted three ICUs with a participation of less than five people per unit from the sample [34]. Descriptive statistics and correlations are reported in Tables 1 and 3, respectively.

### Longitudinal relationships between teamwork, emotional exhaustion, and clinician-rated patient safety

**Fig. 2:**
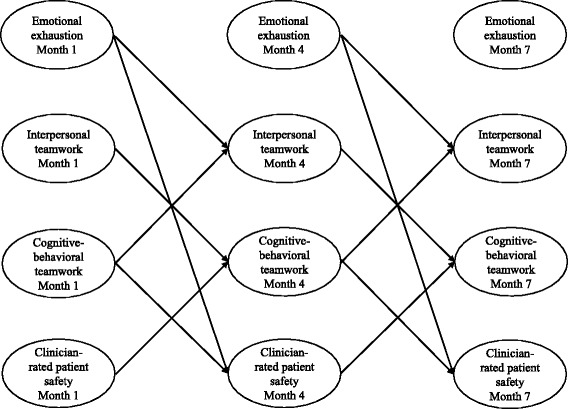
Cross-lagged structural equation model showing significant (reverse) cross-lagged effects (statistics reported in Table 4)

Figure 2 shows the significant cross-lagged and reverse cross-lagged effects of the structural equation model. Analyses revealed that cognitive-behavioral teamwork, interpersonal teamwork, emotional exhaustion, and clinician-rated patient safety were interrelated. Cognitive-behavioral teamwork (β = .17, *p* = .03), but not interpersonal teamwork (β = .03, *p* = .30) predicted an increase in clinician-rated patient safety (see Table 4). In turn, clinicians’ safety perceptions predicted an increase in cognitive-behavioral teamwork (β = .08, *p* = .03).

**Table 4:** Standardized estimates of the structural coefficients in the model

	Outcome			
	Cognitive-behavioral teamwork	Interpersonal teamwork	Emotional exhaustion	Clinician-rated patient safety
Predictor				
Professional role	.08^***^ (.02)	.15^***^ (.02)	.04^*^ (.01)	.06^**^ (.02)
Cognitive-behavioral teamwork	.63^***^ (.03)	.13^***^ (.03)	-.01 (.02)	.17^***^ (.03)
Interpersonal teamwork	.09^**^ (.03)	.56^***^ (.32)	-.03 (.02)	.03 (.30)
Emotional exhaustion	-.01 (.02)	-.07^**^ (.02)	.82^***^ (.01)	-.05 (.09)
Clinician-rated patient safety	.08^**^ (.03)	-.01 (.02)	.02 (.02)	.50^***^ (.03)

Estimates were constrained to be equal across time (e.g., effect of interpersonal teamwork at month 1 on emotional exhaustion at month 4 was set to be equal to the effect of interpersonal teamwork at month 4 on emotional exhaustion at month 7) to increase the reliability and validity of the estimates. Standard errors are in brackets. Model fit indices: RMSEA (root mean square error of approximation) = 0.05, CFI (comparative fit index) = 0.96, TLI (Tucker-Lewis Index) = 0.93, indicating a good fit [48, 49]

^*^ *p* < .05 (two-tailed test); ^**^ *p* < .01 (two-tailed test); ^***^ *p* < .001 (two-tailed test)

Moreover, there was a reciprocal lagged relationship of cognitive-behavioral teamwork on interpersonal teamwork (β = .13, *p* = .03) and vice versa (β = .09, *p* = .03). Thus, cognitive-behavioral teamwork predicts an improvement in interpersonal teamwork and interpersonal teamwork predicts an improvement in cognitive-behavioral teamwork.

Cognitive-behavioral teamwork (β = -.01, *p* = .02) and interpersonal teamwork (β = -.03, *p* = .02) had no effect on later emotional exhaustion. However, emotional exhaustion predicted a deterioration of the quality of interpersonal teamwork (β = -.07, *p* = .02). In addition, there was a tendency for emotional exhaustion to predict a decrease in clinician-rated patient safety (β = -.05, *p* = .09).

In general, physicians reported better cognitive-behavioral teamwork (β = .08, *p* = .02), interpersonal teamwork (β = .15, *p* = .02), clinician-rated patient safety (β = .06, *p* = .02), and higher emotional exhaustion (β = .04, *p* = .01) than nurses.

## Discussion

This study highlights the importance of longitudinal research approaches to examine the complex causal interrelations between teamwork, clinician emotional exhaustion, and patient safety in intensive care settings. Overall, our results suggest that emotionally exhausted clinicians are less able to contribute to effective teamwork, which in turn is necessary to maintain patient safety. Specifically, our analyses showed that low clinician emotional exhaustion increased the quality of interpersonal teamwork. Interpersonal teamwork had a positive effect on cognitive-behavioral teamwork and vice versa. Finally, cognitive-behavioral teamwork positively affected clinician-rated patient safety.

The current study goes beyond prior research that tended to focus on isolated aspects of the multidimensional construct of teamwork [35]. Generally, previous cross-sectional studies addressed team *cognitions and behaviors* in relation to patient safety, and *interpersonal* teamwork aspects in association with clinician emotional exhaustion or burnout in general [35, 36]. We investigated the effect of cognitive-behavioral and interpersonal teamwork on emotional exhaustion *and* patient safety.

### Background

Based on the job demands-resources model, we assumed that effective teamwork may act as a job resource (i.e., aspects of a job that can buffer work demands, achieve work goals, and foster employee well-being) that positively affects patient safety [37].

According to the conservation of resources theory’s (COR) gain cycles, individuals or teams who initially possess plenty of resources are more likely to gain additional resources [38]. Positive interpersonal teamwork may act as a resource fostering the exchange of safety-relevant information between professions, thus increasing patient safety. Similarly, positive cognitive-behavioral teamwork may act as a resource that leads to procedures being carried out more smoothly, fewer errors, and thus higher patient safety. Effective cognitive-behavioral teamwork may be fostered by a good safety climate, which has been shown to be associated with fewer self-reported errors [39]. Teamwork as a resource may also buffer the impact of daily stressors and thus prevent clinician emotional exhaustion.

Lastly, we expected clinician emotional exhaustion to have a negative effect on patient safety. Emotional exhaustion can develop in individuals whose resources are insufficient to meet the cognitive, emotional, or physical demands of their job [39, 40]. Emotionally exhausted clinicians are less vigilant; their motivation to exhibit safe work practices may decrease; and thus errors are more likely to occur [41, 42].

### Differential associations of interpersonal and cognitive-behavioral teamwork with clinician emotional exhaustion and clinician-rated patient safety

Our results showed that cognitive-behavioral teamwork had a positive effect on clinician-rated patient safety, but it was not related to clinician emotional exhaustion. Tangible cognitive-behavioral team processes are instrumental to complete patient care-related tasks, such as pooling collective expertise to solve problems. These processes contribute to higher patient safety, and clinicians may be required to participate in such team processes despite being emotionally exhausted. However, they are not required to invest resources into interpersonal relationships, which might explain the finding that interpersonal teamwork suffered as a consequence of high clinician emotional exhaustion.

Interpersonal teamwork, a global evaluation of the quality of interprofessional collaboration, had no immediate impact on clinician-rated patient safety. It did, however, facilitate cognitive-behavioral teamwork, which in turn positively affected clinician-rated patient safety.

The results highlight the importance of longitudinal studies that go beyond mere cross-sectional associations between teamwork, patient safety and emotional exhaustion, but actually test assumptions concerning causal directions between these constructs.

### Reciprocal relationships between interpersonal and cognitive-behavioral teamwork

Furthermore, our findings demonstrate that the interpersonal and cognitive-behavioral dimensions of teamwork are mutually dependent: On the one hand, better teamwork between professions facilitates cognitive-behavioral teamwork, such as coordination, communication, and cognitive functioning. This is in line with previous work suggesting that interpersonal teamwork forms the foundation on which cognitive-behavioral teamwork components are executed [2]. Trust and mutual respect foster a positive team climate that encourages individuals to contribute their expertise to the common goal, to speak up and voice their concerns in situations where they might deviate from the majority, or to report errors [42]. On the other hand, effective cognitive-behavioral teamwork improves the interpersonal quality of teamwork between professions.

### Effects of emotional exhaustion on patient safety

Our analyses showed that clinician emotional exhaustion and patient safety do not evolve independently. First, our results suggest that emotional exhaustion may have a direct effect on clinician-rated patient safety. These findings complement a previous cross-sectional study conducted in intensive care units showing that emotional exhaustion was not only related to clinician-rated patient safety, but also to standardized mortality ratios [43].

Second, our results imply that clinician emotional exhaustion and clinician-rated patient safety are connected via the reciprocal relationships between interpersonal teamwork and safety organizing: clinicians with low emotional exhaustion may possess more resources to invest in interprofessional relationships. These, in turn, facilitate cognitive-behavioral teamwork and vice versa. Finally, cognitive-behavioral teamwork contributes to higher clinician-rated patient safety.

### Interprofessionalism

Finally, our results highlight the importance of including multiple professions when investigating teamwork. We confirmed that nurses’ and physicians’ ratings of teamwork, emotional exhaustion, and clinician-rated patient safety differ. Previous survey studies that investigated relationships between teamwork and clinician emotional exhaustion or patient safety rarely included multiple professions or explicitly addressed interprofessional teamwork, particularly in intensive care settings [44]. However, interprofessionalism is a defining feature of teams. Our study shows that even in highly technical environments, such as intensive care, the quality of interprofessional collaboration is an important aspect of teamwork that complements the cognitive-behavioral dimension.

### Limitations

The results of this study should be interpreted with some limitations in mind. We focused on the emotional exhaustion subscale from the Maslach Burnout Inventory because the sample size on the unit level did not allow for testing a more complex model that included the depersonalization and reduced personal accomplishment subscales [24]. Nevertheless, we believe that our results are representative and reliable. Emotional exhaustion is the core dimension of burnout and the most reliable and valid subscale across languages and cultures [45]. Moreover, 55 out of 81 Swiss ICUs and a total of 2100 clinicians constitute a high participation rate and large sample size at the individual level.

For economic reasons, patient safety was measured with a single-item indicator that assessed clinicians’ perceptions of overall safety in their unit and may therefore be less reliable than detailed surveys or objective indicators. However, previous research has shown that subjective safety ratings are indicative of objective patient safety such as standardized mortality ratios, as subjective and objective safety measures partly overlap [46]. In addition, our data show a high level of agreement regarding patient safety between team members. This illustrates that safety perceptions are a unit attribute and not only an individual rating impacted by emotional exhaustion and associated negative cognitions. In this study, we focused on developing a general understanding of the causal relationships between two aspects of teamwork, emotional exhaustion, and patient safety. Future research might include a multi multifaceted faceted conceptualization of patient safety into this model.

### Practical implications

Interpersonal and cognitive-behavioral aspects of teamwork build upon one another and are thus both important for effective team functioning. Even in high-technology environments such as the ICU setting, good interpersonal relationships can facilitate cognitive-behavioral teamwork. Thus, interventions targeting teamwork should be designed with both teamwork aspects in mind, as such interventions carry the potential to reinforce each other: inclusion of the entire, multi-professional team; focusing on similarities and shared goals; building of shared mental models; and improving communication and coordination.

Observational studies in intensive care settings have shown the significance of cognitive-behavioral teamwork for immediate team performance outcomes [2]. Our study complements these findings by highlighting long-term effects. Long-term investment in teamwork is likely to build routine on which team members can rely in stressful situations. Previous research has shown that burnout (including emotional exhaustion) can spread from one intensive care clinician to another [47]. It is thus important to prevent the development of clinician emotional exhaustion before it becomes a problem for the entire team, as emotionally exhausted clinicians are less likely to possess the resources to engage in or benefit from team trainings.

## Conclusions

To our knowledge, this is the first study to investigate simultaneous relationships between teamwork, clinician emotional exhaustion, and clinician-rated patient safety using an interprofessional sample. Our results highlight the importance of longitudinal studies, which are necessary to detect long-term, causal effects. Targeting clinician emotional exhaustion is essential in order to ensure effective teamwork and a high level of patient safety. Interventions intended to reduce clinician emotional exhaustion may set a cycle in motion that increases patient safety via mutual reinforcement of interpersonal and cognitive-behavioral teamwork.

### Ethics approval and consent to participate

Ethics permission was granted from the university and the cantonal ethics committees (75, 2013-06-03; 024/13-CER-FR, 2013-24-06). Written consent to participate was obtained per unit from the unit leaders. Upon accessing the online survey, participants were asked for their consent to participate, and assured complete anonymity and confidential handling of their data.

## Abbreviations

CFI: comparative fit index
COR: Conservation of Resources theory
FIML: full information maximum likelihood
HSOPSC: Hospital Survey of Patient Safety Culture
ICU: intensive care unit
M: mean
MBI-HSS: Maslach Burnout Inventory - Human Services Survey
NWI-R: Nursing Work Index - revised
RMSEA: root mean square error of approximation
RN: registered nurse
r_WG_: within-group interrater reliability
SD: standard deviation
SEM: structural equation modeling
TLI: Tucker-Lewis Index

## Footnotes

1 The survey also included the depersonalization and personal accomplishment scales of the Maslach Burnout Inventory [24]. The number of relationships between variables defined in a clustered SEM is limited by the sample size at the unit level. Based on our core research aim of testing simultaneous interrelations and the results of a previous cross-sectional study, which showed that emotional exhaustion was the main predictor of both clinician-rated and objective indicators of patient safety, we opted to exclude the other burnout components in the main analyses to develop a meaningful and reliable statistical model [44].
